# Immunophenotypic features and t(14;18) (q32;q21) translocation of Chinese follicular lymphomas helps to distinguish subgroups

**DOI:** 10.1186/1746-1596-8-154

**Published:** 2013-09-18

**Authors:** Fen Zhang, Li-Xu Yan, Su-Xia Lin, Zi-Yin Ye, Heng-Guo Zhuang, Jing-Ping Yun, Han-Liang Lin, Dong-Lan Luo, Fang-Ping Xu, Xin-Lan Luo, Jie Cheng, Ke-Ping Zhang, Yan-Hui Liu

**Affiliations:** 1 Department of Pathology, Guangdong General Hospital, Guangdong Academy of Medical Science, Guangzhou 510080, China; 2 Department of Pathology, Sun Yat-Sen University Cancer Center, Guangzhou 510060, China; 3 Department of Pathology, the First Affiliated Hospital of Sun Yat-Sen University, Guangzhou 510060, China

**Keywords:** Follicular lymphoma, CD10, BCL6, MUM1, BCL2, Immunophenotype, t(14;18) (q32;q21) translocation

## Abstract

**Background:**

The revised 2008 World Health Organization classification maintains a histological grading system (grades 1–3) for follicular lymphoma (FL). The value of grading FL has been debated. This study will yield deeper insights into the morphologic, immunophenotypic characterization and t(14;18) translocation in FL and explore their significance of diagnosis of Chinese FL subgroups.

**Methods:**

We retrospectively reviewed the FL diagnoses according to the 2008 WHO classification in all diagnostic specimens from a multicentric cohort of 122 Chinese patients. Upon review, 115 cases proved to be truly FL. CD10, BCL6, MUM1, BCL2 and t(14;18) (q32;q21) translocation were detected by Envision immunostaining technique and fluorescence in situ hybridization.

**Results:**

FL1 has larger proportion of follicular pattern (93.0%) than that of FL2 (73.7%, P = 0.036), FL3B (63.6%, P = 0.003) and FL3A (77.4%, P = 0.053), although the last P value was more than 0.05 (Pearson’s chi-squared test). Areas of DLBCL were present in 25.8% (8/31) of FL3A and more frequent in FL3B (59.1%, 13/22; P = 0.015). The positivity of CD10 and BCL2 in FL1-2 were significantly higher than those in FL3 (P < 0.001, P = 0.043, respectively). The positivity of MUM1 in FL1-2 was significantly lower than that in FL3 (10.2% vs. 51.0%; P < 0.001). Furthermore the positivity of MUM1 in FL3A was significantly lower than that in FL3B (37.9% vs. 68.2%; P = 0.032). The positivity of t(14;18) was higher in FL1-2 than in FL3 (73.5% vs. 35.6%, P < 0.001), and was higher in FL3A than in FL3B (51.9% vs. 11.1%, P = 0.005). t(14;18) was significantly correlated with CD10+ (R = 0.453, P < 0.001) and MUM1+ (R = -0.482, P < 0.001).

**Conclusions:**

FL1 and FL2 were immunophenotypically and genomically similar, while FL3A and FL3B were partly immunophenotypically similar but morphologically, genomically distinct. FL3A was genomically closer to FL1-2, whereas FL3A was genomically closer DLBCL. Thus we hypothesize that FL may in fact be a heterogeneous indolent lymphoma encompassing entities with distinct molecular pathogenesis and genetic characteristics. Immunohistochemical and genetic characterization helps to distinguish subgroups of FLs.

**Virtual slides:**

The virtual slide(s) for this article can be found here: http://www.diagnosticpathology.diagnomx.eu/vs/1334018129864616.

## Background

Follicular lymphoma (FL) is an indolent lymphoma. It accounts for 22% of non-Hodgkin’s lymphoma and it is by far the most frequent indolent lymphoma [1]. It is mainly composed of centrocytes with a variable component of centroblasts, the frequency of which gives the grade (1, 2 or 3, depending on the percentage of centroblasts).

Cluster of differentiation 10 (CD10) and B-Cell Lymphoma 6 (BCL6) are biomarker for centrocytes. Tumor cells in typical FL positively express the two or one of the biomarkers. Some cases, especially grade 3B (FL3B), may lack CD10, but retain BCL6 expression [2-4]. Multiple myeloma oncogene 1 (MUM1) is a marker for late germinal center B cells (GCBs) and early post-GCBs [5]. According to our clinical work and reports [3],a few FL are MUM1+. However the significance of expression of MUM1 in FL is still unknown. Most FLs express the anti-apoptotic protein B cell lymphoma 2 (BCL2), while follicles in reactive hyperplasia do not express BCL2. BCL2 positive is an important factor for diagnosis and differential diagnosis of FL.

The t(14;18)(q32;q21) chromosome abnormality is the genetic hallmark of FL. This abnormality juxtaposes the VDJ region of the immunoglobulin heavy chain (*IgH*) gene on chromosome 14q32 with *BCL2* gene on chromosome 18q21, placing BCL2 under the regulatory control of the IgH promotor and causing an overexpression of the BCL2 protein [6]. It is reported [3,7,8] that t(14;18) translocation has been detected in only around 13% of FL3B compared to 58-73% in FL3A and more than 85% of FL1-2. There is no correlation between t(14:18) translocation and BCL2 expression [7].

Several studies have demonstrated that FL exhibits various morphologies, phenotypes, genetic aberrations, and clinical behaviors. These features vary greatly across geographic regions, suggesting geographic heterogeneity as a characteristic of this type of lymphoma. By far, most researches are based on Western population and only a few studies compared the frequency of grade 3A (FL3A) and 3B FL3B cases. Therefore, this study was undertaken to reveal the morphological, immunophenotypic and genetic features of FLs in China. We also investigated the relationship between t(14:18) translocation and the histological grade, as well as the biomarker expression profile of CD10, BCL6, MUM1 and BCL2.

## Methods

### Patients

Biopsy material from 122 unselected cases of FL was retrieved from the surgical pathology and consultation files at Guangdong General Hospital (Guangzhou, China), Sun Yat-sen University Cancer Center (Guangzhou, China), and the First Affiliated Hospital of Sun Yat-sen University (Guangzhou, China) between 2001 and 2010. Archival diagnostic paraffin blocks were available for all cases. The tissue was fixed in 10% neutral buffered formalin, embedded in paraffin, and processed routinely. Four-micrometer sections were stained with H&E for routine histologic evaluation. The histopathology of all cases was reviewed by two of qualified pathologists (Yan-Hui Liu and Heng-Guo Zhuang). Cases were evaluated and graded according to 2008 World Health Organization (WHO) criteria. Upon review, 115 cases proved to be truly FL, and 7 cases were excluded (1 reactive disease, 3 diffuse large B-cell lymphomas, and 3 other types of low-grade B-cell lymphoma). The ethics committee of Guangdong General Hospital & Guangdong Academy of Medical Science approved the study [Reference number No.GDREC2010147H].

### Immunohistochemistry (IHC)

Immunohistochemistry staining was performed using Real Envision Kit (K5007, DAKO, Carpinteria, CA) on an automated immunostaining module (DAKO) according to the manufacturer’s instructions. The antibodies and dilutions employed were as follows: CD10, clone 56C6, 1:100 dilution (Novocastra, Newcastle, UK); BCL2, clone 124, 1:50 dilution (DAKO); BCL6, clone PG-B6p, 1:100 dilution (DAKO); and MUM1, clone MUM1p, 1:400 dilution (DAKO). A negative control was performed in all cases by omitting the primary antibody, which in all instances resulted in negative immunoreactivity. In all cases, internal control was used as positive control.

Only tumor tissues with distinct nuclear staining for BCL6 and MUM1 in >30% of the tumor cells were recorded as positive. Distinct membrane staining for CD10 in > 30% of tumor cells and cytoplasm staining for BCL2 in > 30% of tumor cells was recorded.

### Fluorescence *In Situ* Hybridization (FISH)

FISH for t(14;18) (q32;q21) was performed on formalin-fixed, paraffin-embedded tissue sections as previously described [9] using a dual-fusion LSI IGH/BCL2 probe (08L60-020, Vysis, Downers Grove, IL). FISH signals were analyzed using a fluorescence microscope (Olympus BX51, Tokyo, Japan) equipped with a DP72 camera and DP2-BSW software (Olympus, Tokyo, Japan).

**Figure 1:**
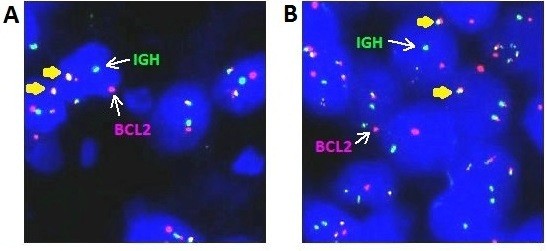
**FISH analysis of t(14;18) (q32;q21) translocation in representative patients with FL (original magnification ×40). (A)** A patients with low-grade FL. **(B)** A patients with high-grade FL. The dual-fusion and dual color IGH/BCL2 probe was used. The expected pattern in a normal nucleus is the two orange, two green signal pattern (2O2G). Fusion signals (yellow arrows) represent fusion genes. In a nucleus harboring a t(14;18), the most common pattern is 1O1G2F.

Tissue sections from twenty reactive lymph node specimens were used to determine the cut-off level for IGH/BCL2 probe. At least 100 intact nuclei per case were evaluated. The mean plus three times the standard deviation (mean + 3SDs) of the normal range was set as the reference range [8]. The signal distribution was evaluated by two independent observers (Fen Zhang and Li-Xu Yan). Translocation of BCL2 was considered positive if they exceeded a cutoff level of more than 5% of the tumor cells (Figure 1).

### Statistical analysis

Statistical analysis was prepared using the Statistical Package of Social Sciences version 18.0 for Windows (SPSS, Chicago, IL). To compare pattern proportions of each histologic grade group, we performed Pearson’s chi-squared test using a R × 2 contingency table. Because the proportions of follicular and diffuse pattern, and focally follicular pattern in each group were small, we analyzed the two groups together as one group. We performed Pearson’s Chi-square test and Fisher’s exact test to compare the incidence of marker expressions and positive t(14;18) in each histologic grade group. Spearman’s rank correlation was used in correlation analysis. Results were considered statistically significant when two-sided *P* < 0.05.

## Results

### Clinical features

The median age of 115 patients with FL at diagnosis was 55 years (range, 22–86 years). Males were more affected than females, with a male-to-female ratio of 1.4:1 (67:48). FL predominantly involved lymph nodes (100/115, 87.0%), but also spleen (3/115, 2.6%) and Waldeyer ring (5/115, 4.3%). The gastrointestinal tract cases involved 3 (2.6%) stomach and 1 (0.9%) ileocecal junction. Occasionally, FL involved parotid (2/115, 1.7%), and thyroid gland (1/115, 0.9%).

### Morphology

Of 115 FLs, 43 (37.4%) were grade 1, 19 (16.5%) were grade 2, and 53 (46.1%) were grade 3. FL1 has larger proportion of follicular pattern (93.0%) than that of FL2 (73.7%, *P* = 0.050 0.036), FL3B (63.6%, *P* = 0.003) and FL3A (77.4%, *P* = 0.053), although the last *P* value was more than 0.05 (Pearson’s chi-squared test). The patterns and grading of 115 FL cases are listed in Table 1. In our study no DLBCL area was found in FL1-2. Areas of DLBCL were present in 25.8% (8/31) of FL3A and more frequent in FL3B (59.1%, 13/22; *P* = 0.015; (Table 2).

**Table 1:** The patterns and grading of 115 FLs

**Patterns**	**Proportion follicular**	**Grading**			
		**FL1 (N = 43)**	**FL2 (N = 19)**	**FL3A (N = 31)**	**FL3B (N = 22)**
		**n (%)**	**n (%)**	**n (%)**	**n (%)**
Follicular	>75%	40 (93.0)	14 (73.7)	24 (77.4)	14 (63.6)
Follicular and diffuse	25-75%	1 (2.3)	2 (10.5)	2 (6.5)	4 (18.2)
Focally follicular/predominantly diffuse	<25%	2 (4.7)	3 (15.8)	5 (16.1)	4 (18.2)

FL, follicular lymphomas; FL1, FL grade 1; FL2, FL grade 2; FL3A, FL grade 3A; FL3B, FL grade 3B.

**Table 2:** The patterns of high-grade FLs

**Patterns**	**High grade**		P*
	**FL3A (N = 31)**	**FL3B (N = 22)**	
	**n (%)**	**n (%)**	
Pure FL3	23 (74.2)	9 (40.9)	0.015
DLBCL with FL3^#^	8 (25.8)	13 (59.1)	

*Pearson’s chi-squared test. ^#^According to 2008 World Health Organization (WHO) criteria, diffuse areas containing >15 centroblasts per high-power field are reported as DLBCL with FL. Pure FL3, FL3 with 100% follicular pattern. DLBCL, diffuse large B-cell lymphoma; FL3, FL grade 3; FL3A, FL grade 3A; FL3B, FL grade 3B; High grade, grade 3.

### Immunohistochemical analysis

**Figure 2:**
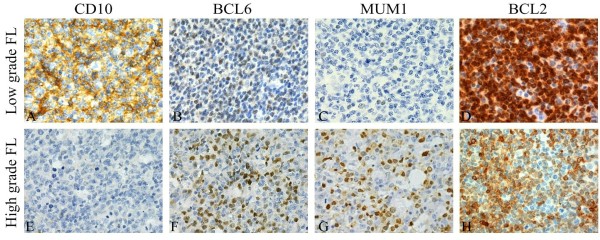
**Immunohistochemical staining of specimens from representative patients with FL (original magnification ×400). (A)** Strong cytoplasmic expression of CD10 in a patient with low-grade FL. **(E)** Tumor cells are negative for CD10 in a patient with high grade FL. **(B, C)** Weak to negative nuclear expression of BCL6 and MUM1 in a patient with low-grade FL. **(F, G)** Partial positivity of tumor cells for BCL6 and MUM1 in a patient with high-grade FL. **(D)** BCL2 protein is expressed with consistent high intensity in the neoplastic cells in low-grade FL. **(H)** BCL2 protein is expressed with inconsistent median to week intensity in high-grade FL, and expressed negatively in some large transformed cells.

Of 115 FLs, CD10 and BCL6 were successfully detected in 114 cases. BCL2 was successfully detected in 111 cases, and MUM1 was detected in 110 cases. CD10 and BCL6 was positive not only at in the tumor follicles, but also at in the interfollicular and diffuse areas of some cases (Figure 2). The staining intensity of CD10 was relatively uniform and the BCL6 staining intensity varies. Both markers expressed stronger in the follicles than in interfollicular and diffuse areas. The positive rate of CD10 in low grade (FL1-2) was significantly higher than that in high grade (FL3; 95.1% *vs.* 66.0%, *P* < 0.001; Figure 2), and it was also higher than that in FL3A (95.1% *vs.* 67.7%, *P* = 0.001 Table 3). There were no statistically significant differences in BCL6 expressions between low grade and high grade, FL1 and FL2, together with FL3A and FL3B (Table 3).

**Table 3:** CD10, BCL6 and MUM1 protein expression the FL subgroups

**FL grading**	**CD10 positive**		**BCL6 positive**		**MUM1 positive**	
	**n/N (%)**	***P***	**n/N (%)**	***P***	**n/N (%)**	***P***
FL1	40/42 (95.2)	1.000^#^	33/42 (78.6)	0.151^#^	3/40 (7.5)	0.376^#^
FL2	18/19 (94.7)		18/19 (94.7)		3/19 (15.8)	
FL3A	21/31 (67.7)	0.756^*^	25/31 (80.6)	1.000^#^	11/29 (37.9)	0.032^*^
FL3B	14/22 (63.6)		17/22 (77.3)		15/22 (68.2)	
FL1-2	58/61 (95.1)	< 0.001^*^	51/61 (83.6)	0.549^*^	6/59 (10.2)	< 0.001^*^
FL3	35/53 (66.0)		42/53 (79.2)		26/51 (51.0)	
FL1-2	58/61 (95.1)	**0.001**^#^	51/61 (83.6)	0.723^*^	6/59 (10.2)	0.002^*^
FL3A	21/31 (67.7)		25/31 (80.6)		11/29 (37.9)	
FL1-3A	79/92 (85.9)	**0.028**^#^	76/92 (82.6)	0.550^#^	17/88 (19.3)	< 0.001^*^
FL3B	14/22 (63.6)		17/22 (77.3)		15/22 (68.2)	
Total	93/114 (81.6)		93/114 (81.6)		32/110 (29.1)	

^*^Pearson’s Chi-square test. ^#^Fisher’s exact test. Bold text indicates significant *P* value. n/N (%), number positive/total number tested (percentage). FL, follicular lymphomas; FL1, FL grade 1; FL2, FL grade 2; FL3A, FL grade 3A; FL3B, FL grade 3B.

The positive rate of MUM1 in low grade was significantly lower than that in high grade (10.2% *vs*. 51.0%, *P* < 0.001; Figure 2, Table 3). Similar difference was found between FL1-2 and FL3A (10.2% *vs*. 37.9%, *P* = 0.002), FL3A and FL3B (37.9% *vs*. 68.2%, *P* = 0.032). However, there was no statistical difference in MUM1 expression between FL1 and FL2 (Table 3).

BCL2 protein was expressed with consistent high intensity in the tumor cells in FL1 and FL2, but with inconsistent median to week intensity in FL3, and negatively in some large transformed cells in FL3 (Figure 2). Positive rate of BCL2 in low grade was higher than that in high grade (96.6% *vs.* 84.6%, *P* = 0.043; Table 4). But no differences were found between other groups.

**Table 4:** BCL2 protein expression and t(14;18) translocation in the FL subgroups

**FL grading**	**BCL2 positive**		**t(14;18) translocation positive**	
	**n/N (%)**	***P*(R)**	**n/N (%)**	***P*(R)**
FL1	38/40 (95.0)	1.000^#^	22/32 (68.8)	0.498^#^
FL2	19/19 (100)		14/17 (82.4)	
FL3A	25/30 (83.3)	1.000^#^	14/27 (51.9)	0.005^*^
FL3B	19/22 (86.4)		2/18 (11.1)	
FL1-2	57/59 (96.6)	**0.043**^#^	36/49 (73.5)	< 0.001^*^
FL3	44/52 (84.6)		16/45 (35.6)	
FL1-2	57/59 (96.6)	0.114^*^	36/49 (73.5)	0.057^*^
FL3A	25/30 (83.3)		14/27 (51.9)	
FL1-3A	82/89 (92.1)	0.412^#^	50/76 (65.8)	< 0.001^*^
FL3B	19/22 (86.4)		2/18 (11.1)	
Total	101/111 (91.0)		52/94 (55.3)	

^*^Pearson’s Chi-square test. ^#^Fisher’s exact test. Bold text indicates significant *P* value. n/N (%), number positive/total number tested (percentage). FL, follicular lymphomas; FL1, FL grade 1; FL2, FL grade 2; FL3A, FL grade 3A; FL3B, FL grade 3B.

Interestingly, data analyses revealed a negative correlation between CD10 and MUM1 protein expression in total cases (n = 110, R = -0.269, *P* = 0.005, Spearman’s rank correlation; Table 5). The proportion of CD10-/MUM1+ in low grade (1/59, 1.7%) was significantly lower than that in high grade (10/51*,* 19.6%; *P* < 0.001). The proportion of CD10-/MUM1+ tended to be lower in FL3A (4/29, 13.8%) compared with that in FL3B (6/22, 27.3%); however, the difference was not statistically significant (*P* = 0.295, Pearson’s chi-squared test).

**Table 5:** Correlation between CD10 and MUM1 protein expression in the FL

		**CD10**		P^*^	R^**^
		**-**	+		
**MUM1**	-	9	69	0.005	-0.269
	+	11	21		

^*^Spearman’s rank correlation. ^**^R, Spearman’s rank correlation coefficient/rho. FL, follicular lymphomas.

### Relationship among histological grades, immunophenotyping and t(14;18) translocation by FISH analysis

t(14;18)(q32;q21) (IGH/BCL2) translocation analysis by FISH was successfully done in 94 of 115 FL cases. Representative positive FISH image are shown in Figure 1. The total positive rate of t(14;18) translocation was 55.3% (52/94). The t(14;18) translocation rate in low grade was much higher than that in high grade (73.5% *vs.* 35.6%, *P* < 0.001). The t(14;18) translocation rate in FL3A was much higher than that in FL3B (51.9% *vs.* 11.1%, *P* = 0.005; Table 4).

The study showed that 60% (54/90) of FL expressed consistent t(14;18) translocation and BCL2 expression, of which 54.4% (49/90) was t(14;18)+/BCL2+ and 5.6% (5/90) was t(14;18)-/BCL2-. The consistent rate of t(14;18) translocation and BCL2 expression decreased with the increase of grading (Table 6). However, no correlations between t(14;18) translocation and BCL2 or BCL6 expression were found (Table 7). t(14;18) translocation was positively correlated to CD10+ (R = 0.453, *P* < 0.001), and negatively correlated to MUM1+ (R = -0.482, *P* < 0.001; Table 7). The t(14;18) translocation rates in CD10-/MUM-, CD10-/MUM1+, CD10+/MUM1- and CD10+/MUM1+ subtypes were 12.5% (1/8), 10% (1/10), 25% (4/16) and 80.4% (45/56), respectively.

**Table 6:** The correlation between FL grading and t(14;18) translocation and BCL2

**FL**	**FL1-2 (%)**	**FL3A (%)**	**FL3B (%)**	**Total (%)**
t(14;18)+/BCL2+	34/46 (73.9)	13/26 (50.0)	2/18 (11.1)	49/90 (54.4)
t(14;18)-/BCL2-	1/46 (2.2)	3/26 (11.5)	1/18 (5.6)	5/90 (5.6)
t(14;18)+/BCL2-	1/46 (2.2)	1/26 (3.8)	0/18 (0)	2/90 (2.2)
t(14;18)-/BCL2+	10/46 (21.7)	9/26 (34.6)	15/18 (83.3)	34/90 (37.8)
Total	46/46 (100)	26/26(100)	18/18 (100)	90/90 (100)

**Table 7:** The correlation of FL immunophenotyping and t(14;18) translocation

**Phenotype**		**Positive t(14;18) translocation**		***P***	**R**
		**n/N**	**%**		
CD10	-	2/19	10.5	< 0.001	0.453
	+	50/75	66.7		
BCL6	-	8/16	50.0	0.605	
	+	44/77	57.1		
BCL2	-	2/7	28.6	0.121	
	+	49/83	59.0		
MUMI	-	46/64	71.9	< 0.001	-0.482
	+	5/26	19.2		

^*^Spearman’s rank correlation. ^**^R, Spearman’s rank correlation coefficient/rho. n/N, number positive/total number tested. -, Negative. +, Positive.

## Discussions

FL is the most common of the indolent non-Hodgkin’s lymphomas, and the second-most-common form of non-Hodgkin’s lymphomas overall. Chinese FL had its unique epidemiological and clinicalopathological features. This study demonstrated a high proportion of FL3 (46.1%), which was very close to a previous report (45.7%) on Chinese cohort [10], but higher than that in western countries [11]. According to a nationwide multicenter study of 10002 cases [12], FL accounts for about 5.5% of all lymphomas in China. The incidence was close to those in Asia and in developing countries, but much lower than those in USA and Western Europe [13,14]. In published studies [15,16], areas of DLBCL are present in 60-80% of FL3B cases and less frequently in FL3A. Our results are very close to those data.

FL is a neoplasm composed of germinal center (GC) B-cells (typically both ceotrocytes and centroblasts cells). FL cells typically express B-cell markers (CD19, CD20, CD22, and CD79a), together with GC-markers (CD10 and BCL6) and BCL2 [11,17]. Some cases, especially FL3B, may lack CD10, but retain BCL6 expression [2-4]. In our FL study CD10 positive rate significantly reduced in high-grade FL compared with low-grade FL, Whereas BCL6 seems to be a more reliable GC-marker than CD10, as it is conserved in higher grade [11].

MUM1 is a marker for late germinal center B cells (GCBs) and early post-GCBs [5]. In our FL series GC-marker CD10 decreased with the increase of FL grade, while post-GC marker MUM1 increased with the increase of FL grade. There were eleven CD10-/MUM1+ cases, of which ten were high grade and one was low grade. The only one low-grade case was morphologically composed of large central cells, and was t(14;18) translocation negative. This was consisted with the previous report that FL composed of large central cells is more aggressive than other low-grade FL, and had lower CD10-positive rate and t(14;18) translocation rate [8]. The proportion of CD10-/MUM1+ cases in CD10- FL rose from 55.0% to 80.0% if the cut-off value for MUM1 positive decreased from 30% to 10%. Hans’ algorithm [18] has been widely used as standard to sub-classify DLBCL into (GCB) and non-GCB origins [19]. If classified according to Hans’s algorithm, 80% (16/20) of CD10- FL were NONnon-GCB subtype and the remaining 20% (4/20) were GCB subtype, indicating possible different origins of CD10-/MUM1+ and CD10-/MUM1- FLs.

BCL2 is a well-known anti-apoptotic protein. Bcl-2 expressed in 91.0% of our FL cases, which falls at the higher end of the reported range of 75-98% [20-22]. Besides, BCL2 protein is expressed by a variable proportion of the tumor cells in 85-90% of cases of low-grade FL, but only 50% of high-grade FL using standard antibodies [22]. Therefore, in clinical work it must be recognized that absence of BCL2 protein dose not exclude FL. In this study, we found that BCL2 was consistently strong in the tumor cells in low-grade FLs, but was inconsistently medium to negative in grade 3 FLs. Whether this is conducive to the diagnosis of high-grade FL needs to be verified by more cases.

The t(14;18) translocation rate in FL1-2 in this study was 73.5%, which was close to Japanese data [23,24], and slightly lower than most of the western reports (80-90%) [25]. This difference may be caused by regional and ethnical differences. The t(14;18) translocation rate in FL3A (51.9%) was close to that in low-grade FL (73.5%). While the rate in FL3B (11.1%) was close to those reported in DLBCL [3] and our previous study [26]. It was reported [24] that FLs without t(14;18) are usually histological FL3B. This may suggest that FLs without the t(14;18) are pathophysiologically distinct from those cases with BCL2 translocation.

The t(14;18)(q32;q21) translocation results in juxtaposition of the IgH and bcl-2 genes, and causes overexpression of BCL2, which prevents cells in follicular center from undergoing apoptosis [27]. However, this study demonstrated no correlation between t(14;18) translocation and BCL2 expression. Absence of BCL2 protein in t(14;18)+/BCL2- cases is partly due to mutations in the *BCL2* gene that eliminate the epitopes recognized by the most commonly used antibody [28]. For t(14;18)-/BCL2+ cases, other molecular mechanisms, for example other types of chromosomal alterations involving the *BCL2* gene on chromosome 18, activation of NF-κB signaling pathway, viral infections, cytokine effects and interactions with cell cycle regulators [6,29]. In this study, the consistent rate of t(14;18) translocation and BCL2 expression decreased with the increase of grading. 83.3% of FL3B were t(14;18) translocation-/BCL2+, suggesting a potential different pathogenesis compared with FL3A (34.6%) and FL1-2 (21.7%).

The value of grading FL has been debated since the 1980s. The 2008 WHO grades classification maintains a histological grading system (grades 1–3) for FL. It has been suggested that the number of grades could be reduced, since FL1 and FL2 are clinically indolent and pathologically similar. Our study revealed that low-grade FL had a high homogeneity in morphology, immune phenotype and t(14;18) translocation. FL3A and FL3B showed significant heterogeneity in t(14;18) translocation and MUM1 expression, although both types showed certain homogeneity in morphology and immunophenotype (CD10, BCL2 and BCL6). Consistent with previous studied [16,30], FL3A was genetically similar to low-grade FL, while FL3B was more similar to DLBCL in t(14;18) translocation.

In conclusion, our study revealed that FL1 and FL2 were immunophenotypically and genomically similar, while FL3A and FL3B were partly immunophenotypically similar but morphologically, genomically distinct. FL3A was genomically closer to FL1-2, whereas FL3A was genomically closer DLBCL. CD10- FL was correlated with MUM1+ and t(14;18)-,suggesting its potentially different origin or pathogenesis from other subgroups. Thus we hypothesize that FL may in fact be a heterogeneous indolent lymphoma encompassing entities with distinct molecular pathogenesis and genetic characteristics. Immunohistochemical and genetic characterization helps to distinguish subgroups of FLs.

## Abbreviations

WHO: World health organization; FL: Follicular lymphoma; FL1: FL grade 1; FL2: FL grade 2; FL1-2: Low grade FL; FL3: FL grade 3, high grade FL; FL3A: FL grade 3A; FL3B: FL grade 3B; CD10: Cluster of differentiation 10; BCL6: B Cell Lymphoma 6; MUM1: Multiple myeloma oncogene 1; GCB: Germinal center B cell; BCL2: B cell lymphoma 2; IHC: Immunohistochemistry; FISH: Fluorescence *In Situ* Hybridization; GC: Germinal center.

## Competing interests

The authors declare that they have no competing interests.

## Authors’ contributions

FZ and LY worked on data acquisition and analysis, constructed of tissue microarray, developed the algorithm, and drafted the manuscript; YL contributed to the study design, data acquisition and analysis; HZ contributed to the study design and data analysis; ZY, JY, HL and SL worked on aspects of data acquisition and revision of the manuscript; FX and DL conceived and coordinated the study; XL, JC and KZ were involved in IHC and FISH. All authors read and approved the final manuscript.
